# Separation of the Proximal Humeral Epiphysis in the Newborn: Rapid Diagnosis with Ultrasonography

**DOI:** 10.1155/2015/825413

**Published:** 2015-01-28

**Authors:** Rachelle Goldfisher, John Amodio

**Affiliations:** Department of Radiology, SUNY Downstate Medical Center, 450 Clarkson Avenue, Brooklyn, NY 11203, USA

## Abstract

Separation of the proximal humeral epiphysis (SPHE) is a well-known occurrence and may occur secondary to trauma, infection, and nonaccidental trauma. Since most newborns do not have the proximal humeral epiphysis ossified at birth, the diagnosis may be difficult to make on routine radiographs. Ultrasonography of the shoulder in the newborn is rapid, noninvasive, and nonionizing imaging techniques which can diagnose SPHE. In this report, we describe and emphasize the diagnostic utility of state-of-the-art ultrasonography for the diagnosis of SPHE.

## 1. Introduction

Separation of the proximal humeral epiphysis (SPHE) is a well-known occurrence, and may occur secondary to trauma, infection, and nonaccidental trauma. SPHE is known to exist in the newborn after a traumatic delivery.

Since most newborns do not have the proximal humeral epiphysis ossified at birth, the diagnosis may be difficult to make on routine radiographs. Widening of the glenohumeral distance may be a clue on plain film, but it is not always accurate [1].

Ultrasonography of the shoulder in the newborn is rapid, noninvasive, and nonionizing imaging techniques which can diagnose SPHE. Additionally, it can be performed at the bedside. To the best of our knowledge, there have been few reports in the literature demonstrating SPHE in the newborn period with sonography [2–4]. In this report, we describe and emphasize the diagnostic utility of state-of-the-art ultrasonography for the diagnosis of SPHE.

## 2. Case Report

The patient is an ex 37-week gestational infant who presented with shoulder dystocia. The APGARS at birth were 3, 4, and 7. The infant required positive pressure ventilation and intubation for respiratory distress. Examination in the neonatal ice revealed reduced left arm motion, with swelling, ecchymoses, and tenderness to palpation. Left elbow reflexes were spontaneous and there was a positive left grasp. The infant was not moving the right upper extremity and was diagnosed as having a right Erb's palsy.

**Figure 1:**
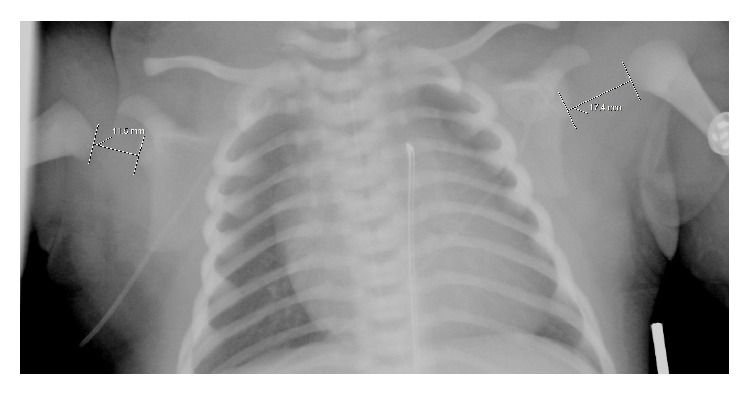
AP view of the chest demonstrates the ossification center of the right humerus is within the glenohumeral joint. The ossification center of the left humerus is not visualized. Additionally, the left glenohumeral distance on the left is increased compared to the right.

**Figure 2:**
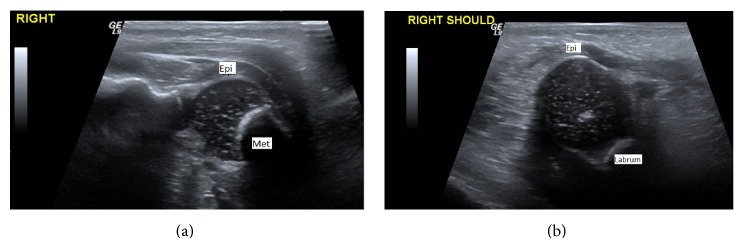
(a) Longitudinal sonogram of the right shoulder demonstrates the normal relationship of the epiphysis (epi) on the metaphysic (met). (b) Transverse sonogram of the right shoulder demonstrated the right humeral epiphysis within the right glenoid labrum.

**Figure 3:**
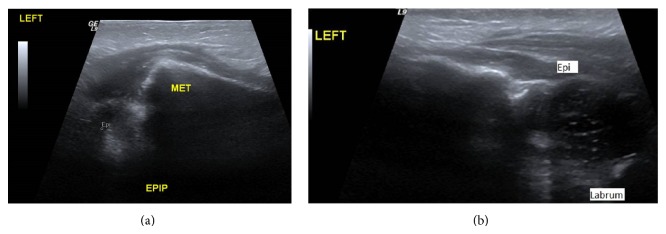
(a) Longitudinal sonogram of the left shoulder demonstrates separation of the left humeral epiphysis (epi) from the metaphysis (met). (b) Transverse sonogram of the left shoulder shows left humeral epiphysis within the confines of the labrum.

**Figure 4:**
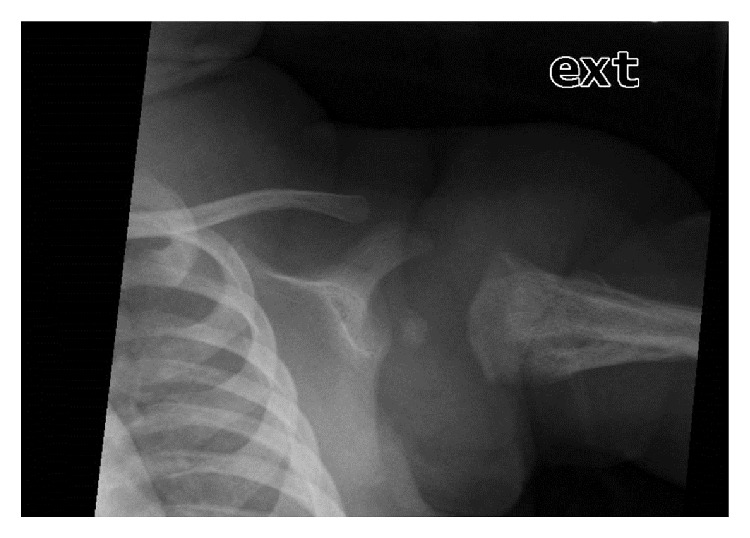
AP view of the left shoulder demonstrates abundant new bone along the left humeral shaft compatible with healing and ossification of the epiphysis within the glenoid labrum.

X-ray examination of the chest demonstrated a mild air space disease pattern, ossification of the right humeral epiphysis, but the ossification center of the left humeral epiphysis was not clearly visualized (Figure 1). Additionally, the left glenohumeral distance on the left was increased compared to the right. A sonogram of both humeri was obtained which demonstrated a normal ephyseal–humeral relationship on the right (Figure 2(a)); the right femoral epiphysis was located within the normal glenoid labrum (Figure 2(b)). The left humeral epiphysis was displaced from the metaphysis, compatible with epiphyseal separation (Figure 3(a)). The left epiphysis was normally located within the glenohumeral joint (Figure 3(b)). The left shoulder was placed in a sling; subsequent X-ray examination demonstrated healing of the epiphyseal separation (Figure 4).

## 3. Discussion

The physeal plate is less resistant to trauma in infants and children than are the joint capsule, bone, and ligaments [1]. Therefore, the path of least resistance of forces applied to the extremities is through the cartilaginous physis. When there is a difficulty delivery, or shoulder dystocia, as in the case presented here, the force applied may be through the proximal humeral physis, resulting in epiphyseal separation from the metaphysis.

Ekengren et al. [4] described 21 infants with 21 epiphyseal separations at birth. Nine of the separations were of the proximal humerus and five of the distal humerus; one of the proximal portion of the femur; five of the distal portion of the femur; and one of the distal portions of the tibia and fibula. Interestingly, according to Lemperg and Liliequist [5] epiphyseal separation of the proximal humerus is rarely accompanied by an injury of the brachial plexus.

Clinically, the infant with an epiphyseal separation of the proximal humerus will move the affected arm little and there is pain and tenderness on physical examination. There may be swelling of the affected region as well as possible hematoma [2].

Broker and Burbach [2] state that the proximal humerus epiphysis ossifies in 15–20% of infants by 39 weeks of gestational age and in 40% at 41 gestational ages. However, the most recent version of Dr. Caffey's textbook [6] states that the proximal humeral ossification center ossifies shortly after birth with 5% and 95% confidence intervals of 37 weeks' gestation and 16 weeks' postnatally, respectively. Nevertheless, the proximal humerus ossification center is not ossified in the majority of full term infants, making plain film evaluation of the injured shoulder quite difficult, if at all possible. A factor which may complicate the diagnosis further is that if the arm is in internal rotation at the time of radiographic examination, the ossification center, if visible, may appear in a more central position in relationship to the humeral shaft, thus giving the false impression of a normal relationship between the epiphysis and the humerus. An asymmetric position of the humeral shaft with the glenohumeral joint, when compared to the unaffected side, may offer a clue to the diagnosis of epiphyseal separation, when the ossification center is not yet ossified. In such cases, the metaphysis may appear more caudad in relation to the scapula.

Ultrasound is a noninvasive, nonionizing radiation examination which can be rapidly performed at the bedside. The major advantage of sonography is that this technique can image the ossification center, even if it is purely cartilaginous. Thus, the relationship of the epiphysis and metaphysic can readily be identified. In addition, the relationship of the ossification center with the glenohumeral joint can also be easily depicted, Figure 2(a).

MRI can also demonstrate the diagnosis of epiphyseal separation, as well as evaluate the brachial plexus and other soft tissue abnormalities [7]. However, MRI requires transporting the patient to the scanner; special coils are needed to maximize imaging of the affected part and may require that the infant be sedated to minimized motion artifact.

Treatment of epiphyseal separation is usually conservative, with immobilization for several weeks. In general, the prognosis for adequate healing and no residual deformity is excellent, as the plan of cleavage is extra-articular, which insures little or no vascular compromise of the epiphysis [2]. Occasionally, closed reduction may fail. El-adl et al. [7] reported eight cases of SPHE which failed closed reduction; subsequent reduction was performed using k-wires, with subsequent excellent healing and no avascular necrosis of the epiphysis or limb-length deformity.

In summary, SPHE should be considered in the differential diagnosis of the neonate with birth trauma who has limitation of motion and tenderness of the arm. The diagnosis may be difficult to make on the basis of plain radiography. Ultrasonography is a rapid bedside examination which can accurately make the diagnosis.
